# Post-acute COVID-19 syndrome and its prolonged effects: An updated systematic review

**DOI:** 10.1016/j.amsu.2022.103995

**Published:** 2022-06-15

**Authors:** Talal Almas, Jahanzeb Malik, Abdulla K. Alsubai, Syed Muhammad Jawad Zaidi, Raafe Iqbal, Kashif Khan, Muhammad Ali, Uzma Ishaq, Majid Alsufyani, Sebastian Hadeed, Reema Alsufyani, Reema Ahmed, Tushar Thakur, Helen Huang, Meetty Antony, Ishan Antony, Anhad Bhullar, Farida Kotait, Lubabah Al-Ani

**Affiliations:** a Royal College of Surgeons in Ireland, Dublin, Ireland; b Department of Cardiology, Rawalpindi Institute of Cardiology, Rawalpindi, 46000, Pakistan; c Department of Medicine, Rawalpindi Medical University, Rawalpindi, 46000, Pakistan; d Department of Cardiology, Pakistan Ordinance Factories Hospital, Wah, 47040, Pakistan; e Department of Hematology, Foundation University Medical College, Rawalpindi, 46000, Pakistan; f National University of Ireland—Galway, Galway, Ireland; g Jawaharlal Nehru Medical College, Belgaum, India

**Keywords:** COVID-19, Long COVID, SARS-CoV-2

## Abstract

**Objective:**

This systematic review aimed at estimating the prevalence of post-acute COVID-19 symptoms in view of published literature that studied prolonged clinical manifestations after recovery from acute COVID-19 infection.

**Methods:**

Relevant databases were searched for extraction of articles. For data synthesis, based on the distribution of quantitative variables, they were expressed as mean ± standard deviation (SD) or median and interquartile range (IQR). Qualitative variables were presented as frequency (n) and percentages (%).

**Results:**

Twenty-one articles qualified for the final analysis. The most common persistent clinical manifestations were fatigue (54.11%), dyspnea (24.38%), alopecia (23.21%), hyperhidrosis (23.6%), insomnia (25.98%), anxiety (17.29%), and arthralgia (16.35%). In addition to these symptoms, new-onset hypertension, diabetes, neuropsychiatric disorders, and bladder incontinence were also reported.

**Conclusion:**

Clinical features of post-acute COVID-19 infection can manifest even after 60 days of initial infection. Multidisciplinary care along with regular follow-up must be provided to such patients.

## Highlights

• Clinical features of post-acute COVID-19 infection can manifest even after 60 days of initial infection.
• Prolonged symptoms and signs are being reported in observational studies and case reports every day. Although such symptoms are usually experienced in survivors of critical illness, the post-acute effects of COVID-19 are equally being reported in patients with mild severity of disease who do not require hospitalization.
• Necessary future research includes stratification of these post-acute effects with gender, age, and comorbid conditions in acute, subacute, and chronic phases of the disease.

## 1 Introduction

Severe acute respiratory syndrome coronavirus 2 (SARS-COV-2) presented as clustered cases of atypical pneumonia in the city of Wuhan in the Hubei province of China. In March 2020, coronavirus disease 2019 (COVID-19) was declared as a pandemic by World Health Organization (WHO) and since then approximately 148 million people have been infected with the virus [1]. There is a well-established pool of scientific knowledge about the acute effects of COVID-19 and unprecedented efforts of the scientific community have now shifted towards the long-lasting sequelae of the disease, effects of which are yet to be seen [[2], [3], [4], [5]].

The term “long COVID” was used in social media to indicate persistence of symptoms after weeks or months of recovery from SARS-COV-2 infection. It is also called “post-acute COVID-19 syndrome” due to its remitting and relapsing nature. There can be persistence of one or more symptom or appearance of new symptoms. As most patients with post-acute COVID-19 syndrome are PCR negative, it indicates that there is microbiological recovery. However, there is a time lag between microbiological recovery and clinical recovery. There are several barriers in diagnosing post-acute COVID-19 because the time for clinical recovery varies with severity of illness; while associated complications make it difficult to define the cut-off time for the diagnosis.

Prolonged symptoms and signs are being reported in observational studies and case reports every day [6]. Although such symptoms are usually experienced in survivors of critical illness, the post-acute effects of COVID-19 are equally being reported in patients with mild severity of disease who do not require hospitalization [7]. Therefore, this systematic review was conducted to estimate the prevalence of persisting COVID-19 signs and symptoms after recovery.

## 2 Methods

### 2.1 Search strategy

A protocol for the selection of articles and carrying out the systematic review of the literature was made after a consensus among the authors and subject experts, but it was not deposited in a registry. Data was collected after protocol approval from the ethical review board of Foundation University Medical College (ID#FFH/ADC/021/21).

**Fig. 1:**
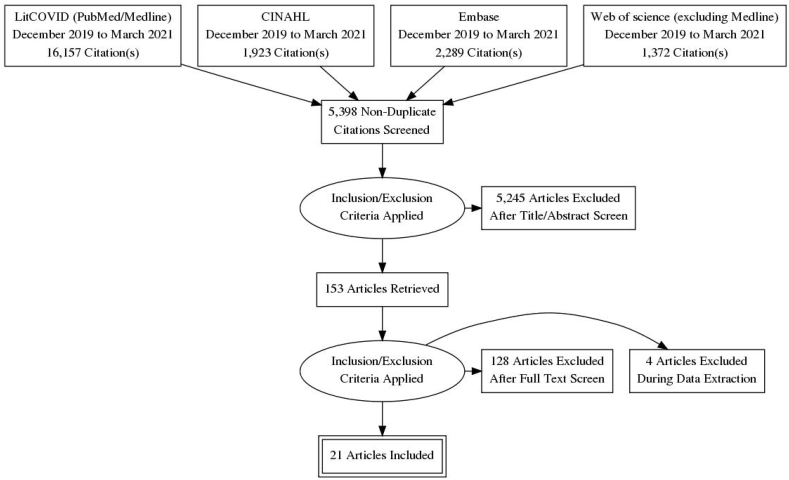
PRISMA flow chart.

The search terms used in the search strategy were as follows: (((“long-haul” coronavirus disease OR post-acute COVID-19 OR “convalescent” COVID-19) OR prolonged coronavirus infection OR coronavirus disease [Mesh]) OR “severe acute respiratory syndrome coronavirus 2” chronic disease [Supplementary Concept]) OR recurrent OR lingering OR complications of “COVID-19” [Mesh] OR “betacoronavirus” [Mesh])) AND 2019/12 [PDAT]: 2030 [PDAT]))). The systematic review followed the Preferred Reporting Items for Systematic Reviewers and Meta-analysis (PRISMA) guidelines and the PRISMA flowchart is demonstrated in Fig. 1 [9].

### 2.2 Selection criteria

The main databases used for study selection were PubMed and Medline through LitCOVID (accessed on 17th April 2021) [8], the Cumulative Index to Nursing and Allied Health Literature (CINAHL) (accessed on 12th April 2021), Embase, and Web of Science (accessed on 12th April 2021). Articles published before 1st May 2021 were included in the search. We included randomized clinical trials, observational, cross-sectional, and cohort studies which were in the English language, and peer-reviewed published articles that reported signs and symptoms after at least two weeks from the recovery of acute COVID-19 in adults. Investigations on children (<18 years) were excluded. Only studies with more than 50 participants were included. Post-acute COVID-19 syndrome was defined as symptomatology after two weeks of recovery from COVID-19.

### 2.3 Data extraction

Pre-prints, case reports, editorials, and data notes were excluded. After the initial search and removal of duplicates, all the search was imported on EndNote version 20 (Clarivate Analytics™). All the screening and inclusion of the articles were conducted by two independent reviewers (TA, SMJZ) blinded to each other's decisions. Once the initial screening was finished, all the included studies were referenced in Mendeley.

The two reviewers (TA, SMJZ) reviewed full texts for final inclusion. Where there was a dispute, a third reviewer (MA) resolved it between them. The descriptive variables extracted were country, setting, follow-up time, sample size, mean age and percentage of gender, outcomes, symptoms, and signs, and names used for post-acute COVID-19 syndrome. No automation tool was used.

### 2.4 Data synthesis

For statistical analysis, Statistical Package for the Social Sciences (SPSS) version 26 (IBM Corp. Armonk, NY, USA) was used, and based on the distribution of quantitative variables, they were expressed as mean ± standard deviation (SD) or median and interquartile range (IQR). Qualitative variables were presented as frequency (n) and percentages (%).

### 2.5 Risk of bias assessment

All included articles were assessed using the Newcastle-Ottawa Scale (NOS) [10]. Scales are provided resources 1 and 2.

## 3 Results

A total of 21,741 titles and abstracts were screened for this review. Of these, 153 full texts were reviewed and according to the review protocol, 56 were excluded because of inappropriate sample size, 47 presented acute COVID-19 symptoms, 23 were case series, and 6 excluded as data notes. A total of 21 studies were included for final analysis and review and their general characteristics are shown in Table 2. Many studies assessed a specific long-term symptom after COVID-19 recovery and the PRISMA flowchart for study selection is presented in Fig. 1. A total of 10 studies were from Europe and three from the United States. Others were from Mexico, Saudi Arabia, China, Australia, and Bangladesh. All studies included were on either previously hospitalized or non-hospitalized patients, and most of them had mild, moderate, and severe states of COVID-19 patients. Total follow-up time was more than one month in the majority of the studies and the number of the patient cohort was 54,730 participants with a median age of 54 years. Except for two studies, there was no stratification among gender differences between post-COVID-19 symptoms.

**Table 1:** Newcastle-Ottawa Scale assessment of pooled studies, * = one score 0 = no score.

Studies	Selection				Comparability	Outcomes			Total
	Representativeness of exposed cohorts	Selection of non-exposed cohorts	Ascertainment of exposure	Outcome not present at the start of the study		Assessment of outcomes	Length of follow-up	Adequacy of follow-up	
Cardi et al.	*	0	*	*	0	*	*	*	******
Mandal et al.	*	0	*	*	0	*	*	*	******
Chopra et al.	*	0	*	*	0	*	*	*	******
El Sayed et al.	*	0	*	*	0	*	*	*	******
Mahmud et al.	*	0	*	*	0	*	*	*	******
Carvalho-Schnieider et al.	*	0	*	*	0	*	*	*	******
Marva et al.	*	0	*	*	0	*	*	*	******
Galván-Tejada et al.	*	*	*	*	**	*	*	*	*********
Moreno-Pérez et al.	*	0	*	*	0	*	*	0	*****
Halpin et al.	*	0	*	*	0	*	*	*	******
Huang et al.	*	0	*	*	0	*	*	*	******
Xiong et al.	*	0	*	*	0	*	*	0	*****
Tenford et al.	*	0	*	*	0	*	*	*	******
Taquet et al.	*	0	*	*	0	*	*	*	******
Townsend et al.	*	0	*	*	0	*	0	0	****
Garrigues et al.	*	0	*	*	0	*	*	*	******
Horvath et al.	*	0	*	*	0	*	*	*	******
Arnold et al.	*	0	*	*	0	*	*	*	******
Osikomaiya et al.	*	0	*	*	0	*	*	*	******
Leth et al.	*	0	*	*	0	*	*	0	*****
Sudra et al.	*	0	*	*	0	*	*	0	*****

**Table 2:** General article characteristics and study population. Standard deviation (SD), interquartile range (IQR).

#	Author [ref]	Country	Setting	Follow-up (number of days)	Study participants	Sample size (n)	Age; mean ± SD/median (IQR)	Males; %	Outcome variables
1	Carfì et al. [14]	Italy	Single-centered	60	Patients meeting the following criteria (no fever for 3 consecutive days, improvement in symptoms, and 2 negative test results for SARS-CoV-2 virus 24 h apart)	143	56.5 **±** 14.6	62.9%	Quality of life assessment after acute COVID-19, length of hospital stay, Number of persistent symptoms. Fever, fatigue, red eyes, chest pain, cough, anosmia, dysgeusia, myalgia, diarrhea
2	Mandal et al. [16]	London, United Kingdom	Multi-centric (3 hospitals)	45	Patients with abnormal blood tests or imaging at discharge.	384	59.9 ± 16.1	62%	Symptom persistence including breathlessness, cough, fatigue, and, poor sleep quality. Laboratory parameters including TLC, platlet count, Lymphocyte count, D dimers, LFTs, and CRP levels
3	Chopra et al. [21]	United States	Multi-centric (38 hospitals)	60	ICU/Hospitalized COVID-19 patients discharged between 16 March and July 1, 2020 at 38 hospitals.	488	62 (50–72) years	51.8%	Mortality and rehospitalization, Primary care follow-up, New/worsened symptoms, Return to normal activity, Emotional impact, Financial loss/impact
4	El Sayed et al. [17]	Saudi Arabia	Single centered	14	Patients of COVID-19 after 2 consecutive negative PCR tests attending pulmonology clinic for follow-up	200	36.58 ± 9.85	57%	Assessment of fatigue and anhedonia using validated scales.
5	Mahmud et al. [6]	Dhaka, Bangladesh	Single centered	30	Discharged COVID-19 patients	355	39.8 ± 13.4	58.3%	The frequency and interval of a spectrum of post COVID-19 symptoms were assessed. These include post viral fatigue, persistent cough, insomnia, Circadian rhythm sleep disorders, headache, vertigo, Post-exertional dyspnea, rash, pneumonia, restless leg syndrome, chest pain, Adjustment disorder, Nasal blockage, Excessive sweating, Disturbance of memory, New-onset diabetes or hypertension, myalgias, and Precipitation of gout
6	Carvalho-Schneider et al. [28]	France	Single centered	60	Post COVID-19 patients with or without clinical signs of pneumonia but without a need for oxygen therapy (mild/moderate disease)	150	49 ± 15 years	44%	Persisting symptoms at Day 30 and 60 which included Fever, dyspnea, chest pain, abnormal auscultation, flu-like symptoms, digestive disorders, weight loss, anosmia, palpitations, arthralgia, cutaneous rashes
7	Marwa et al. [36]	Egypt	Single centered	14	Patients recovered from COVID-19	287	32.3 ± 8.5	35.8%	Fatigue, anxiety, joint pain, continuous headache, chest pain, dementia, depression, dyspnea, blurred vision, tinnitus, intermittent fever, obsessive compulsive disorder
8	Galván-Tejada et al. [32]	Mexico	Multi centeric	14	Cases: Patients who had a laboratory-confirmed diagnosis of SARS-CoV-2, and in whom at least fourteen days have passed since the appearance of symptoms. Controls: Patients with no laboratory or clinically proven COVID-19 infection	141 cases and 78 controls. (Total 218)	Means of 39.14 years for females and 39.01 for males respectively	49%	Fever, myalgia, rhinorrhea or coryza, asthenia, cough, cephalgia, red eyes, odynophagia, nausea, vomit or diarrhea, anosmia or dysgeusia, stomach pain or discomfort, dyspnea, chills
9	Moreno-Pérez et al. [18]	Spain	Single centric	98	Hospitalized Patients who had laboratory proven SARS-COV-2	277	56.0 (42.0–67.5)	52.7%	Post- COVID syndrome. These include pneumonia, fatigue, anosmia, dyspnea, persistent cough, headache fever, diarrhea, neurological symptoms, and laboratory features
10	Halpin et al. [22]	United Kingdom	Single centered	30–60	Hospitalized Patients who had laboratory proven SARS-COV-2 and were discharged from hospital	100	For ward patients: 70.5 (20–93) For ICU patients: 58.5 (34–84)	54%	Fatigue, Breathlessness, Neuropsychological symptoms, Speech and swallowing problems, weight loss/gain, bowel/bladder incontinence, Perceived health, quality of life, and Vocation change since COVID‐19 illness.
11	Huang et al. [7]	China	Single centered	186	patients with laboratory confirmed COVID-19 who were discharged between Jan 7, and May 29, 2020	1733	57·0 (47·0–65·0)	52%	Fatigues, sleeping problems, hairloss, anosmia, palpitations, joint pain, decreased appetite, taste disorder, chest pain, myalgias, rashes, swallowing difficulty, Low grade fever, eGFR, and quality of life
12	Xiong et al. [27]	China	Single centered	90	All COVID-19 survivors who were diagnosed with COVID-19 according to WHO interim guidance and were discharged from the hospital by March 1, 2020	538	52.0 (41.0–62.0) years	45.5%	Fatigue, swelling, myalgias, arthralgia, chills, limb edema, dizziness, chest pain, post activity polypnea, cough sputum, throat pain, palpitations, discontinuous flushing, new onset hypertension, depression, anxiety, and alopecia
13	Tenforde et al. [35]	United States	Single centered	14–21	adults aged ≥18 years who had a first positive RT-PCR test for SARS-CoV-2, and reported persistence COVID-19 symptoms	270	26% patients aged between 18 and 34 years, 32% aged between 35 and 49 years, and 47% aged ≥50 years	48.14%	Risk Factors for Delayed Return to Usual Health Among COVID-19 patients were evaluated. The outcome variables included age, comorbids, ethinicity, gender
14	Taquet et al. [24]	United States	Multicentric, electronic records	14–90 days	Discharged COVID-19 patients with no previous psychiatric illness	44,779	49.3 (19.2)<	45.1%	New onset psychiatric illness disorders psychotic, insomnia, mood disorders (depressive episodes) anxiety disorders (PTSD, panic disorder, adjustment disorder and generalized anxiety disorder).
15	Townsend et al. [15]	Ireland	Single centered, outpatient clinic	56–84	Mild, moderate Symptomatic patients and Hospitalized patients	128	49.5 ± 15 years	46.1%	Persistent fatigue
16	Garrigues et al. [37]	France	Single centered	110	Discharged COVID-19 patients who were Hospitalized in ward or ICU	120	63.2 (15.7) years	62.5%	Cough, chest pain, fatigue, dyspnea, ageusia, anosmia, hair loss, attention disorder, memory loss, sleep disorder
17	Horvath et al. [38]	Australia	Multicentric, computed records	83	Discharged COVID-19 patients with mild to moderate disease intensity	102	45 (17–87) years	40%	Smell reduction, taste change, cough, fever, headaches, worsening nasal blockage, runny nose, fatigue, sore throat.
18	Arnold et al. [39]	United Kingdom	Single centered	28	patients (≥18years of age) admitted with COVID-1	110	60 (46–73) years	56%	Fever, cough arthralgia, myalgias, chest pain, anosmia, diarrhea, abdominal pain, headache, insomnia, deranged blood tests, spirometry and chest C ray
19	Osikomaiya et al. [40]	Nigeria	Multi- centered	14	Discharged COVID-19 patients who were Hospitalized in ward or ICU	274	41.8 ± 11.8 years	66.1%	Fever, fatigue, weight loss, malaise, cough, dyspnea, chest pain, anosmia, loss of appetite, dizziness, palpitations, insomnia vertigo, dysgeusia
20	Leth et al. [41]	Denmark	Single centered	84	Hospitalized COVID-19 that were discharged after negative PCR	71 patients	58 (48–73)	43%	Difficulty in concentration, paresthesia's, headache, anosmia, taste impairment, cough dyspnea, expectoration, sore throat,
21	Sudre et al. [23]	United Kingdom	Multi-center	90	Mobile health app users with PCR positive COVID-19 patients/negative matched controls	4182	44 (28, 56)	28.5%	Number of symptoms, duration of symptoms, quality of life,

The general quality of the studies was assessed using the Newcastle-Ottawa scale for observational studies (Table 1). There were 35 post-acute symptoms and signs presented in the study cohort of these articles. They are stratified in Table 3. The most common manifestations were fatigue (54.11%), dyspnea (24.38%), alopecia (23.21%), hyperhidrosis (23.6%), insomnia (25.98%), anxiety (17.29%), and arthralgia (16.35%). Thirteen studies reported fatigue and anosmia, 15 dyspnea, 12 chest pain, and 11 non-productive coughs, and 5 studies showed more than one symptom. Apart from constitutional symptoms of COVID-19, personality and sleep disorders, bladder and bowel incontinence, new-onset hypertension, and diabetes were also seen in the included studies.

**Table 3:** Post-acute COVID-19 signs and symptoms after recovery (pooled prevalence, %).

Clinical characteristics of post-acute COVID-19	Studies (n)	Number of patients with symptoms (n)	Total number of patients (n)	Pooled Prevalence; %
Fatigue	13	2412	4457	54.11%
Hyperhidrosis	1	127	538	23.6%
Migraine-like Headache	8	221	3006	0.03%
Vertigo	2	11	629	1.74%
Alopecia/Telogen effluvium	3	537	2313	23.21%
Dyspnea	15	790	3242	24.38%
Anosmia	13	497	3924	12.66%
Dry eyes	1	21	146	14.38%
Blurred vision	3	70	838	8.35%
Dysgeusia/Ageusia	7	293	3009	9.73%
Arthralgia	5	198	1211	16.35%
Myalgias	9	204	3527	5.78%
Adjustment disorder	1	2	355	0.56%
Anxiety	3	160	925	17.29%
Dementia	1	82	287	28.57%
Dizziness	3	123	2467	4.98%
Depression	2	117	825	14.18%
Cough	11	434	2527	17.17%
Expectoration	1	16	538	2.97%
Insomnia	6	725	2790	25.98%
Obsessive compulsive disorder	1	14	287	4.87%
New-onset hypertension	3	10	1180	0.84%
New-onset diabetes	2	3	642	0.46%
Palpitations	5	252	2952	8.53%
Discontinuous flushing	1	26	538	4.83%
Restless leg syndrome	1	2	355	0.56%
Pedal edema	1	14	538	2.6%
Memory disturbances	4	69	849	8.12%
Rash/Cutaneous signs	4	87	2417	3.59%
Chest pain	12	459	4422	10.37%
Sore throat/odynophagia	6	150	2886	5.19%
Bowel/bladder incontinence	1	8	100	8%
Tinnitus	1	48	287	16.72%
Weight loss	3	42	504	8.33%
Diarrhea/Vomiting/gastrointestinal issues	7	236	2911	8.1%
More than one symptom	5	456	1533	29.74%

## 4 Discussion

The most important pathophysiological mechanism in acute COVID-19 is direct viral toxicity leading to endothelial damage and microvascular injury [11]. This can cause immune system dysregulation and hyperinflammatory states, hypercoagulability, and downregulation of the angiotensin-converting enzyme 2 (ACE2) pathway [11]. In contrast to this, the post-acute effects of COVID-19 are an overlap of phylogenetic similarities with SARS-COV-1 and Middle-Eastern respiratory syndrome (MERS) viruses [12]. However, SARS-COV-2 has a higher affinity for ACE2 compared with SARS-COV-1, and this mechanism may be the contributing factor in the widespread transmission of SARS-COV-2. Furthermore, potential mechanisms behind post-acute symptoms and signs in COVID-19 recovered patients seem multifactorial: the pathophysiologic changes caused by the virus, inflammatory and immune-mediated cell damage, and sequelae of recovery from critical illness [13].

This systematic review demonstrated that 68% of patients have at least one post-acute symptom after recovery from COVID-19. A total of 21 studies were included in this review which fulfilled our inclusion criteria and overall, 35 signs and symptoms of post-acute COVID-19 were identified in this cohort of patients (Table 3). The most common symptoms were fatigue, dyspnea, hyperhidrosis, dementia, depression, alopecia, and cough. The majority of presenting symptoms or signs were similar to the acute presentation of COVID-19. However, a possibility remains for other effects to be identified later in this pandemic. In the following discussion, we will elaborate on the most common symptoms and signs of post-acute COVID-19 to understand each disease in more detail.

Overall, the most common symptom among all the included patients was the feeling of tiredness or fatigue (54.11%) [6,7,[14], [15], [16], [17], [18]]. It was present after three months’ follow-up in critical COVID-19 patients admitted to intensive care units (ICUs) [15]. This phenomenon has been established in survivors of critical illness (post-ICU syndrome), even after years of recovery, where half the patients report symptoms of chronic fatigue syndrome, including incapacitating fatigue, generalized body pain, neurocognitive disturbances, insomnia, and increased sympathetic drive [19]. Viruses like Epstein-Barr virus, cytomegalovirus, and herpes virus have been implicated in causing chronic fatigue syndrome and this review adds SARS-COV-2 as the causative agent of chronic fatigue [20].

Neuropsychiatric symptoms are also reported in some studies, including headache, insomnia, anxiety, depression, bladder and bowel incontinence, ageusia, migraine, and dementia [6,16,[21], [22], [23], [24]]. Similar to chronic fatigue syndrome, the etiology, and pathophysiology of neuropsychiatric symptoms in COVID-19 are multifactorial and unclear. In a cohort of 355 patients in Bangladesh, and 143 patients in Italy, a cumulative 63% of the patients were screened positive in at least one of the domains evaluated for neuropsychiatric sequelae (depression, anxiety, insomnia, obsessive-compulsive disorders, etc.) [6,14]. Clinical depression and anxiety were reported in approximately 17% of patients following COVID-19 [6]. Memory loss in the form of dementia and ageusia is also reported in a few studies, including cognitive impairment with or without fluctuations [25]. All these symptoms could be related to the social stigma of contracting a potentially fatal illness, some effects of sedatives in critical COVID-19 patients with delirium, and hypercoagulability leading to cerebrovascular disease. In addition, post-recovery sleep disturbances can also precipitate psychiatric disorders [26]. Mental health assessment and mental health attention models are very important in the post-acute COVID-19 stage, as they can contribute to a better quality of life in this cohort. Telogen effluvium and alopecia are also reported in three studies, which is defined as temporary hair loss due to excessive shedding of Telogen hair after COVID-19. Although self-limiting, this condition can cause emotional distress in many patients [27].

Dyspnea (24.38%) and cough (17.17%) were the most prominent pulmonary symptoms in this review [28]. Several studies have demonstrated persistent high resolution computed tomography (HRCT) lung abnormalities after 60 days from the initial presentation [29]. In addition, previous studies have exhibited lung dysfunction in more than 50% of the patients compared to our study cohort [7,30]. A decreased diffusion capacity due to loss of lung volume is the most commonly reported pathophysiologic impairment in post-acute effects of COVID-19, which is directly related to the severity of acute illness [31,32]. This observation is consistent with SARS and MERS and seems to be the contributing factor in long-term pulmonary sequelae of COVID-19. There is the viral-dependent invasion of endothelial-epithelial barrier causing infiltration of monocytes and macrophages, leading to extravasation of protein-rich exudate filling the alveolar space. This is similar to acute respiratory distress syndrome (ARDS) [33]. There are reports of pulmonary vascular micro and macrothrombosis in 20% of the patients with critical COVID-19 pneumonia and the severity of the endothelial injury and widespread microangiopathy seen on lung histopathology is greater than that seen in ARDS from other viruses [34,35].

Several other constitutional symptoms are demonstrated in this review [[36], [37], [38], [39], [40], [41]]. The most important of them are weight loss, new-onset diabetes and hypertension, expectoration, blurred vision, and dry eyes. Chest pain is reported in up to 10% of COVID-19 survivors at 60 days follow up, while ongoing palpitations were reported in 8.53% at 6-months follow up. Apart from acute coronary syndrome (ACS) and myocarditis, an increased incidence of takotsubo cardiomyopathy is being reported in this pandemic compared with the pre-pandemic period (7.8% vs. 1.5%, respectively) [42]. Mechanisms contributing to cardiovascular sequelae in post-acute COVID-19 seem to be downregulation of ACE2 and renin-angiotensin-aldosterone system (RAAS), cytokine storm-related deterioration of myocardial integrity, pericarditis, and arrhythmias [43].

A recent meta-analysis identified studies assessing the long-term effects of COVID-19. They included 15 studies and estimated that 80% of the infected patients with SARS-COV-2 developed one or more long-term symptoms [44]. One other living systematic review included 39 studies and showed weakness (41%), general malaise (33%), and fatigue (31%) as the most commonly occurring symptoms [45]. Similarly, our estimated that 68% of the patients developed one or more symptoms after COVID-19 recovery with fatigue, dyspnea, and dementia as the most common symptoms.

This systematic review had several limitations. One is the small number of studies with underpowered sample size, creating a potential bias and variation in defined outcomes leading to the heterogeneity of the results. Many studies used a self-reporting method which can produce an interobserver bias and almost all studies enrolled COVID-19 patients in mild, moderate, and severe disease category with variable follow-up times references. This can produce heterogeneous results. There was a predefined assessment of symptoms in every study assessed, which can lead to unreported outcomes. Although high viral load is implicated in the long-term sequelae of COVID-19, there is no definition of the effect of late effects of COVID-19 and its associated symptoms. A critical illness survivor can have prolonged symptoms while a patient with mild disease can recover early from the same problem. Hence, there is a need for prospective studies to determine if the post-acute COVID-19 effects are a continuation of SARS-COV-2 or complications of premorbid conditions.

### 4.1 Future directions

The provision of post-hospital discharge care of COVID-19 patients is an evolving field and may differ across institutions. The current mainstay of treatment involves the use of dexamethasone and antivirals along with early rehabilitation interventions during the post-hospitalization stage, with management largely dictated by the severity of the disease. Therefore, clinicians who are meticulously reporting and managing those afflicted with the syndrome have a crucial role in the future towards creating the appropriate protocols and management plans that will significantly improve patient outcomes. Moreover, studies and active research are required to optimise the management of post-hospital discharge care of COVID-19 patients.

## 5 Conclusion

The multiorgan sequelae of SARS-COV-2 infection beyond the acute infection are increasingly being recognized with an increasing clinical experience and pool of data becoming available rapidly on COVID-19. This updated systematic review of 21 studies and 54,730 patients is the largest cohort of patients with post-acute effects of COVID-19 evaluated to date. It demonstrated that post-acute effects of COVID-19 can persist even at six months and from the clinical point of view, medical professionals should look for the symptoms and signs in patients recovered from COVID-19. Necessary future research includes stratification of these post-acute effects with gender, age, and comorbid conditions in acute, subacute, and chronic phases of the disease. This will lead to a better understanding of the delayed sequelae of COVID-19. Through this review, it is clear that acute care of COVID-19 does not conclude at hospital discharge, and interdisciplinary care is needed for comprehensive care of these patients at homes and outpatient clinics. Hence, healthcare systems must establish dedicated COVID-19 clinics, where specialists from various disciplines can provide unanimous care.

## Ethical approval

NA.

## Sources of funding

NA.

## Author contribution

TA, JM; concept, literature search, first draft, final draft, methodology, analysis; AKA, SMJZ; literature review, study selection, first draft; RI; first and final draft; KK; first draft, supervision, methodology; FK, FK, LA; literature search, first draft; MA, RA; literature search, first draft; MA, SH, ASR, TK; first draft, analysis, literature search; AUW, TT, RA, IA, MA; first and final draft, methodology; UI; supervision, concept; final draft, analysis.

## Please state any conflicts of interest

NA.

## Registration of research studies

1. Name of the registry: NA
2. Unique Identifying number or registration ID: NA
3. Hyperlink to your specific registration (must be publicly accessible and will be checked): NA

## Guarantor

Dr. Jahanzeb Malik and Dr. Talal Almas.

## Consent

Written and informed consent was obtained and is available to the editor in chief upon request.

## Provenance and peer-review

Not commissioned, externally peer-reviewed.

## Funding

Authors did not receive any specific funding for this article.
